# Observations on the Angustura Bark

**Published:** 1790

**Authors:** Augustus Brande

**Affiliations:** Apothecary to the Queen


V. *Obſervations on the Anguſtura Bark. Communicated in a Letter to Dr.* Simmons, *F. R. S. by* Auguſtus Brande, *Eſq. Apothecary to the Queen.*

THE Anguſtura bark, of which I believe the firſt accounts appeared in the London Medical Journal *, differs ſo much from every

* Vol. X. pages 154 and 158.

drug drug known to me, that I had tried it, and made ſome experiments upon it, before I ſaw thoſe papers. I have ſince frequently given this bark; and as varied and numerous trials can alone enable us to form a true judgment of the real merits of a medicine, ſo I ſhould hope it may be of ſome uſe to point out the caſes in which it has appeared of ſervice, and to endeavour more nearly to aſcertain its proper doſe than has hitherto been done.

The Anguſtura bark contains a warm bitter, readily taken up by boiling, or even by cold water; an acrid reſin; and ſome eſſential oil. Of extract it yields a large proportion, altogether nearly one half its weight. That which is prepared with water only is a not unpleaſant bitter. The reſiduum, digeſted with rectified ſpirit, gives a deep tincture, leaving, on evaporation, a dark-coloured reſin, very acrid and nauſeous. The tincture, made with one ounce of this bark to ſixteen ounces of proof-ſpirit, appears to poſſeſs moſt of its powers. The powder is of a brightiſh yellow colour, nearly reſembling rhubarb.

To eight ounces of the decoction, or infuſion, (prepared with three or four drachms of the bark to ſixteen ounces of water) I have generally nerally added half an ounce of ſyrup of orange peel and a drachm of compound tincture of lavender. Theſe additions make the medicine more pleaſant, without, I ſhould ſuppoſe, influencing its effects.

Of the powder I give ftom ten to twenty grains, and of the decoction, or infuſion, from an ounce to an ounce and a half for a doſe.

Large doſes of this bark have appeared to me not to agree, nor indeed to act, ſo well. Moderate doſes ſit eaſy on the ſtomach, and give rather a grateful ſenſation of warmth. In delicate habits, however, twenty grains have, in one or two inſtances, produced ſome nauſea.

Having repeatedly taken this bark myſelf, I can confirm what is ſaid in Dr. Williams's paper*, that it is, in general, leſs apt to occaſion coſtiveneſs, and a diſagreeable ſenſe of weight and fulneſs, than the Peruvian bark. This, together with the ſmallneſs of the doſe, would make it the more eligible medicine for weak ſtomachs, or where we may apprehend obſtructions of the viſcera. Indeed it has, when given under ſuch circumſtances as a tonic, agreed apparently better.

* See Vol. X. page 159.

How

How far the Anguftura may merit to be placed in competition with the Peruvian bark, as a febrifuge, cannot perhaps as yet be decided. I have myfelf had no opportunity of trying it in agues; but a very refpectable friend informs me that he has fucceeded, and with very fmall dofes. One tertian was ftopped by fix dofes of fifteen grains each. A fecond, which had continued three weeks, yielded to two fuch dofes. Both have, after two months, had no return. In other intermittent complaints, and in two cafes of low fever, I have found this bark of great fervice.

Having myfelf fuffered repeatedly and very feverely from aguifh pains in the teeth and face, I found that taking one drachm of Peruvian bark, at the firft approach of pain, and repeating the dofe after half an hour, if neceffary, would infallibly, and often inftantaneoufly, remove the whole complaint, and prevent its confequences. Accident induced me to fubftitute the Anguftura bark, of which I chewed perhaps ten or fifteen grains, with the fame good effect. This attack was fevere, and for two or three days I felt fome flight returns; but chewing a little more of this bark, and taking a dofe or two of the tincture, carried them off completely, and I continued well near three months, an interval unuſually long, the ſeaſon conſidered. In January laſt a repetition of the remedy became neceſſary. The effect was the ſame; and where I have given it to others in the intervals or remiſſions from pain, it has rarely failed of affording relief.

Againſt diarrhœas, and even dyſenteries, I almoſt conſider this bark as poſſeſſed of ſpecific powers. A woman, turned of forty, and of a bad habit of body, was, in October laſt, ſeized with a dyſentery. She had been under my care the autumn before for the ſame complaint, which then gave me much trouble, and left her very weak. I had at that time adviſed her, in caſe of any return, to begin with a laxative, inſtead of aſtringents, as ſhe had done; and accordingly three doſes of Glauber's ſalts had now been taken before I ſaw her, which, however, afforded no permanent relief, every dyſenteric ſymptom continuing with conſiderable violence. I gave her ten drachms of the Anguſtura infuſion, with three drops of laudanum, at firſt, every two or three hours; but finding her much better the following day, the opiate was omitted, and the infuſion only given three or four times a day. About the third day ſhe had

ſome

ſome return of pain and teneſmus, which were, however, ſoon removed by a gentle laxative and continuing the infuſion.

Two other caſes have ſince occurred, which were treated nearly in the ſame manner, only no opiate was given; and, after evacuating, I truſted wholly to the Anguſtura infuſion and an occaſional gentle laxative. Both my patients were much relieved in a few hours, and recovered completely in the courſe of three or four days.

In diarrhœas the infuſion has afforded almoſt conſtant and ſpeedy relief. It generally has been given alone, after premiſing a gentle laxative; but I have, where there has been ſome feveriſh heat, added half an ounce of ſaline mixture to each doſe, and the effect has been the ſame. The laxative has commonly been repeated a day or two after the purging had ſtopped.

To a patient, whoſe diarrhœa had continued upwards of a twelvemonth, and reduced him greatly, I gave the tincture twice a day with ſucceſs, though his manner of life by no means aſſiſted the cure. It was, altogether, taken ſome weeks, as he had ſeveral relapſes from getting wet.

The following cafe, which is ftrongly in favour of the bark, has been kindly communicated to me by Dr. Willan :

" G. S., a labouring man, aged forty years, " had been fubject to a diarrhœa upwards of " three months. He had from eight to twelve " ftools every day. The difcharge was mu- " cous and frothy, and not confiderable in " quantity. It was always attended with much " pain. At other times he had frequent gri- " pings and tenefmus. At the latter part of " the period mentioned the ftools were ufually " tinged with blood. He got very little reft, " and was very much reduced by the com- " plaint fo long continued.

" Laxatives, opiates, fmall dofes of ipeca- " cuanha, &c., were prefcribed for him with " little advantage.

" I then ordered half a fcruple of the cortex " Anguftūræ to be taken three times a day ; " the quantity of the powder was gradually " increafed to a fcruple, which dofe produced " no naufea.

" The frequency of the difcharges, the pain " and tenefmus, gradually abated under this " ufe of the medicine, and at the end of three " weeks he was perfectly recovered."

I once

I once tried the Anguftura bark in a colliquative diarrhœa attendant on the laft ftage of hectic fever, but without effect: indeed, as it rather increafed the fever, I did not perfift.

The general and often fudden good effects of this bark in diarrhœas appear the more remarkable, as it betrays no aftringency to the tafte, nor does it ftrike a purple colour with vitriol of iron.

As a tonic and ftomachic I have frequently and fuccefsfully given the Anguftura bark; and occafionally as an alterative in fcrophulous habits, and in thofe eruptions which are commonly termed fcorbutic. Other powerful medicines having, in thefe latter cafes, been combined with it, the fhare of praife due to the bark cannot well be determined; yet, though the dofes were much fmaller, it anfwered equally with Peruvian bark, in the room of which it was ufed.

To conclude—the Anguftura bark appears to me, and to fome of my medical friends, to be poffeffed of powers which give it ftrong claims to farther trial; and I would therefore hope that thofe who are folicitous for the improvement of phyfic, and better enabled to decide on its real merits, may confider it as not unworthy of their attention.

*Arlington Street,*
February 8th, 1790.

